# Editorial: Recent advances in surgical management of NSCLC

**DOI:** 10.3389/fonc.2024.1454905

**Published:** 2024-08-08

**Authors:** Marcello Migliore, Domenico Galetta, Masayuki Chida

**Affiliations:** ^1^ Thoracic Surgery, King Faisal Specialist Hospital Research Centre, Organ Transplant Centre of Excellence, Riyadh, Saudi Arabia; ^2^ Department of Surgery and Medical Specialties, University of Catania, Catania, Italy; ^3^ Thoracic Surgery, San Giovanni Bosco Hospital, Turin, Italy; ^4^ General Thoracic Surgery, Dokkyo Medical University, Mibu, Japan

**Keywords:** VATS, individualization, NSCLC, precision surgery, sublobar, artificial intelligence, AI

Lung cancer remains a leading cause of cancer-related deaths worldwide, and non-small cell lung cancer (NSCLC) accounts for approximately 80% of all lung cancers. Despite advances in therapy, surgical resection remains the cornerstone of treatment for early-stage NSCLC.

Over the past few years, there have been significant advances in the surgical approach and operative techniques for the management of NSCLC. After decades where lobectomy was the most common operation performed for early NSCLC, more and more signals suggest that the approach for T1N0 NSCLC has shifted to segmentectomy or wedge resection (1–8).

This Research Topic collected 12 publications: four original research papers, one brief research report, three systematic reviews, two reviews, and two case reports. The Research Topic is a valuable resource and platform for thoracic surgeons and oncologists, with the aim of improving outcomes for lung cancer patients, and provides a comprehensive overview of recent advances in the surgical management of NSCLC.

Various aspects of NSCLC management, including surgical approaches (RATS, VATS multiportal or uniportal) and techniques, perioperative care, and outcomes are presented in the Research Topic.

Song et al. compared survival after lobectomy and wedge resection for stage IA second primary NSCLC (SP-NSCLC) patients with previous lung cancer-directed surgery using overall survival (OS) and lung cancer-specific mortality as outcomes. The authors showed a 5-year overall survival (OS) was 61.3% with wedge resection and 66.1% with lobectomy. They concluded that wedge resection is comparable to lobectomy in OS for stage IA SP-NSCLC patients with previous lung cancer-directed surgery. In short, this paper shows that wedge resection may be sufficient for stage IA SP-NSCLC.

Ding et al. showed that wedge resection plus adequate lymph nodes resection was comparable to lobectomy for small-sized non-small cell lung cancer. The authors identified patients diagnosed with node-negative NSCLC ≤2 cm who underwent wedge resection or lobectomy (2004-2015). Then patients were stratified by the procedure (wedge resection or lobectomy) and the size of NSCLC (≤1 cm, 1-2 cm). For lesions ≤1 cm and receiving lobectomy, lymph nodes resection had no impact on survival. Wedge resection and lobectomy were comparable when one or more nodes for lesions ≤1 cm and six or more nodes for lesions 1-2 cm were resected.

Lu et al. conducted a meta-analysis based on randomized controlled trials comparing lobectomy and sublobar resection for stage I non-small cell lung cancer. Their systematic analysis included five RCTs and 2222 patients. The authors showed no statistical difference in OS (HR=0.87, p=0.445) and DFS (HR=0.99, p=0.918) between patients who underwent lobectomy and sublobar resection during the total follow-up period. The strong findings led the authors to suggest that lobectomy is usually not a justified operation for stage I NSCLC. 

Although retrospective, the articles of Song et al., Ding et al., and Lu et al. are significant because the results are similar to those of the prospective randomized trial performed by Altorki et al. (9, 10). A question arises. What could happen in the future on the basis of the results of their studies? Their results suggest that minimal lung resection, even a wedge, will be sufficient to guarantee long-term survival in early-stage NSCLC patients and therefore larger resection, such as segmentectomy or even lobectomy, will be performed less. It will take years to change practice worldwide, but it will certainly happen.

Pan et al. evaluated, through a propensity score-matched comparison, the results of robotic- and video-assisted thoracoscopic surgery and open lobectomy (OL) for non-small cell lung cancer patients aged 75 years or older. The authors reported that RATS possessed superiority in better perioperative outcomes over VATS and OL in very old NSCLC patients.

Huang et al. performed a systematic review and meta-analysis of prospective studies comparing robot-assisted thoracic surgery to video-assisted thoracic surgery. Of 614 patients, 299 patients were treated by RATS and 315 by VATS. In the RATS group, blood loss was lower (P = 0.009) and more nodes stations were dissected in RATS (P < 0.001). Nevertheless, no significant difference occurred between RATS and VATS in length of hospital stay, readmission, operative time, conversion, number of dissected lymph nodes, upstaging rate, time of chest tube drainage, or post-operative complications. The authors concluded that, except for the higher total cost, RATS has obvious advantages in lymphadenectomy and bleeding.

What is important after reading these two articles comparing RATS with VATS is that surgeons should not confound the approach with the operation. In fact, as expected, the different surgical approaches achieved comparable survival outcomes. This is due to the fact that, although different approaches have been used, the surgeon inside the chest performs an identical operation (lung resection and lymphadenectomy), and therefore survival is the same. This result confirms that the surgeon should use the approach that suits them best to enhance treatment of the patient (11).

Gallina et al. analyzed the predictive factors of unforeseen nodal metastases in resected clinical stage I NSCLC. With a total of 297 patients, the authors showed a significant correlation with the upstaging rate. This result was confirmed in the multivariate analysis with an OR= 2.545 (p=0.02) for the number of resected lymph nodes and an OR=2.717 (p=0.01) for the high-grade pattern of adenocarcinoma. The Italian group have shown that, in patients with clinical stage I NSCLC, the number of resected lymph nodes and the histological subtype of adenocarcinoma can be significantly associated with nodal metastasis. Certainly in the future, this result will encourage thoracic surgeons to perform a better and more extended lymphadenectomy.

Abbaker et al. wrote an interesting and comprehensive review of the current state of artificial intelligence (AI) applications in lung cancer management. In the preoperative phase, AI enhances diagnostics and predicts molecular biomarkers, especially in cases with limited biopsy materials. Intraoperatively, AI transforms surgery by providing real-time guidance and decision support. Postoperatively, AI aids in pathological assessment and predictive modeling for refined care. AI could certainly be of help when interpretability is difficult and different opinions arise between physicians and surgeons on how to conduct a treatment strategy. Although the role of 3D reconstruction in thoracic surgery and artificial intelligence is at its beginning, there are a lot of expectations for the future. We believe that there is a need for clearer indications because, at the moment, there are no well-defined guidelines and confusion exists.

Kamigaichi et al. discussed the indications of segmentectomy, especially for patients with radiologically pure-solid NSCLC. Although radiologically pure-solid NSCLC, lacking ground-glass opacity (GGO) components, could represent highly malignant neoplasm with poor prognoses compared to those containing GGO components, the subgroup analysis of the JCOG0802/WJOG4607L proved the efficacy of segmentectomy for pure-solid NSCLC. Recently, the JCOG1211 demonstrated the efficacy of segmentectomy even for NSCLC measuring up to 3 cm with GGO predominance and for some tumors measuring > 2–3 cm. The authors expect that segmentectomy may become an appropriate treatment modality for radiologically pure-solid NSCLC of 2–3 cm because of the survival benefits associated with the lung-sparing approach. However, the benefits of segmentectomy for patients with pure-solid NSCLC of 2-3 cm must be confirmed by future clinical trials.

Zhang et al. made an interesting review on the clinical application of three-dimensional (3D) technology in video-assisted thoracoscopic surgery sublobectomy and its future direction. It is evident that a more frequent use of 3D technologies in locating pulmonary nodules and identifying variations in target lung segmental vessels and bronchi play pivotal roles in VATS sublobectomy, especially in preoperative planning, intraoperative navigation, and doctor-patient communication.

Li et al. described the detailed classification of the interlobar artery and the artery crossing intersegmental planes in the right upper lobe, which is useful during segmentectomy. The authors demonstrated over 600 cases in which variation types of blood vessels in the right upper lobe were complex. This article could help thoracic surgeons understand anatomy variations, accurately locate lesions before surgery, and effectively plan surgeries.

Although the role of 3D reconstruction in thoracic surgery is well known, it also has the potential to help surgeons to know the anatomical variation of pulmonary vessels or bronchi preoperatively and to demonstrate intersegmental planes to perform precise operations such as segmentectomy; it is also useful for intraoperative navigation. In the future it will certainly be used more.

Finally, the Research Topic includes two very interesting case reports.

Tombelli et al. reported their experience with three successful left tracheal sleeve pneumonectomies and one neocarina reconstruction surgery for benign lesions without lung resections and without cardiovascular support such as cardiopulmonary bypass. These three case reports confirm the good long-term survival demonstrated in previous experiences on tracheal sleeve pneumonectomy after induction therapy (12, 13). Wu et al. reported uniportal video-assisted (3) thoracoscopic segmentectomy for a low-grade type fetal adenocarcinoma patient with poor pulmonary function.

In conclusion, while reading the papers presented in this Research Topic, a significant positive correlation between sublobar resection and long-term survival in NSCLC less than 2 cm was found. The results suggest that lobectomy will probably be less used in the upcoming years for initial-stage lung cancers. Although wedge resection seems appropriate for peripheral lung tumors, its significance for individualizing treatment is still a source of discussion. Artificial Intelligence and 3D technology will contribute to the modern revolution in the practice of thoracic surgery. Other areas of research which have not been discussed in depth in this Research Topic are immunotherapy (4), the treatment of ground-glass opacities (5, 6), and the treatment of locally advanced lung cancer (7). Furthermore, it appears evident that, in the future, individualization of surgery (8) for NSCLC would be the more appropriate approach to achieving long-term survival.
